# The dual role of BI 2536, a small-molecule inhibitor that targets PLK1, in induction of apoptosis and attenuation of autophagy in neuroblastoma cells

**DOI:** 10.7150/jca.33110

**Published:** 2020-03-05

**Authors:** Zhiheng Li, Chun Yang, Xiaolu Li, Xiaojuan Du, Yanfang Tao, Junli Ren, Fang Fang, Yi Xie, Mei Li, Guanghui Qian, Lixiao Xu, Xu Cao, Yi Wu, Haitao Lv, Shaoyan Hu, Jun Lu, Jian Pan

**Affiliations:** 1 Institute of Pediatric Research, Children's Hospital of Soochow University, Suzhou 215003, China;; 2 Department of Gastroenterology, The 5th Hospital of Chinese PLA, Yinchuan, Ningxia, China;; 3 Department of Hematology and Oncology, Children's Hospital of Soochow University, Suzhou 215003, China.; 4 Department of Pediatric Surgery, Children's Hospital of Soochow University, Suzhou 215003, China.; 5 Department of Pathology, Children's Hospital of Soochow University, Suzhou 215003, China.; 6 Department of Cardiology, Children's Hospital of Soochow University, Suzhou 215003, China.

**Keywords:** BI 2536, apoptosis, autophagy, neuroblastoma, polo-like kinase 1 (PLK1)

## Abstract

Neuroblastoma (NB) is the most common extra-cranial solid tumor in childhood with the overall 5 years' survival less than 40%. Polo-like kinase 1 (PLK1) is a serine/threonine-protein kinase expressed during mitosis and over expressed in multiple cancers, including neuroblastoma. We found that higher PLK1 expression related to poor outcome of NB patients. BI2536, a small molecule inhibitor against PLK1, significantly reduced cell viability in a panel of NB cell lines, with IC50 less than 100 nM. PLK1 inhibition by BI 2536 treatment induced cell cycle arrest at G_2_/M phase and cell apoptosis in NB cells. Realtime PCR array revealed the PLK1 inhibition related genes, such as BIRC7, TNFSF10, LGALS1 and DAD1 *et al*. Moreover, autophagy activity was investigated in the NB cells treated with BI 2536. BI 2536 treatment in NB cells increased LC3-II puncta formation and LC3-II expression. Formation of autophagosome induced by BI 2536 was observed by transmission electron microscopy. However, BI2536 abrogated the autophagic flux in NB cells by reducing SQSTM1/p62 expression and AMPKα^T172^ phosphorylation. These results provide new clues for the molecular mechanism of cell death induced by BI 2536 and suggest that BI 2536 may act as new candidate drug for neuroblastoma.

## Introduction

Neuroblastoma (NB) is the most common extra-cranial solid tumor of childhood and accounts for 8% to 10% of childhood cancers and 15% of pediatric oncology death 1, 2. Treatment for neuroblastoma is a multimodality therapy composed of surgery, chemotherapy, autologous stem cell transplant, immunotherapy and radiotherapy either in combination or separately depending on disease stage, patient age, genetic abnormalities, tumor biology, risk group and histological classifications 3. Although outcome for patients with neuroblastoma has improved, approximately 50% of cases are high-risk with overall 5 years' survival rates less than 40% 4. So, it is crucial to call for more effective strategies for NB treatment.

Uncontrolled proliferation is the characteristic of cancer, including neuroblastoma 5. Evidence shows that altered expression of cell cycle genes is the predominant contributor to the aggressive NB phenotype 6. To this end, much interest has turned to the regulator of cell cycle progression to identify new potential targets for anticancer therapies 7. Numerous investigations have now established that the cell cycle is controlled by a series of different kinases that regulate the mitosis progression, such as the cyclin-dependent kinases (CDKs) and polo-like kinases (PLKs) family. In PLKs family, the first identified member polo was shown to be essential to mitosis in Drosophila 8. In humans, five members of PLK family have been identified, that are PLK1-5 9-12. Among them, PLK1 is the best-characterized family member. Studies have showed that the biological role of PLK1 was involved in precise regulation of mitotic progression 13. In cell cycle, the expression and activity of PLK1 is varied, presented as low throughout G_0_, G_1_ and S phase, rising in G_2_ and reaching at a maximal level during M phase, suggesting that PLK1 functions in multiple steps of mitosis 11, 14. PLK1 was widely over expressed in a broad spectrum of human cancers, such as breast cancer 15, colorectal cancer 16, glioma 17, non-small cell lung cancer 18 et al. Also, the elevated expression of PLK1 has prognostic value for predicting poor outcomes in patients in a variety of cancers 19-21. Inhibition or deletion of PLK1 expression resulted in mitotic catastrophe and induced apoptosis in cancer cells 22, 23. Therefore, PLK1 was considered a promising target for anti-cancer drug development.

To date, a large number of small molecule inhibitors against PLK1 have been developed and some of them have been tested in preclinical trial. BI 2536, an ATP-competitive PLK1 kinase inhibitor, has been shown to inhibit PLK1 enzyme activity at nanomolar concentrations 24. Studies showed that BI 2536 treatment resulted in cell cycle progression disorder, mitotic catastrophe, growth inhibition and apoptosis in a broad range of cancer cells. In view of its potent anti-proliferation activity in a variety of cancer cells and xenograft models, the effect of this compound has been evaluated in clinical studies in several adult cancers 25-27.

In neuroblastoma, PLK1 was reported over expressed in high risk NB patients and could be viewed as an unfavorable prognostic marker in NB 28. But until now the role of PLK1 in neuroblastoma has not been fully understood. In this study, we employed BI 2536--- the PLK1 inhibitor ---to assess the role of PLK1 in neuroblastoma and the anti-cancer efficacies of BI 2536 in neuroblastoma cells.

## Materials and Methods

### Cell lines and reagents

Human neuroblastoma cell lines with MYCN amplification (NGP, KELLY, SK-N-BE(2), NGP and KP-N-NS) and without MYCN amplification (SH-SY5Y, SK-N-SH and NBL-S) were obtained from JENNIO Biological Technology (Guangzhou, China) within 5 years. Cells were maintained at 37 °C in a humidified atmosphere with 5% CO_2_ in Dulbecco's modified Eagle's medium (DMEM) or RPMI-1640 medium (Life Technologies, Inc., Darmstadt, Germany), supplemented with 10% fetal bovine serum (FBS) (Atlanta Biologicals, Lawrenceville, GA, USA), penicillin (100 u/ml) and streptomycin (100 µg/ml) (Sigma, St. Louis, MO, USA). BI 2536 was purchased from Selleck Chemicals (Houston, TX, USA). BI 2536 was dissolved in DMSO at a concentration of 10 mM and stored at -80 °C.

### Cell proliferation

NB cells were seeded in 96-well plates at a density of 2x10^4^ cells per well, allowed to attach for 18hr and following various concentrations of BI 2536 treatments. After 24hr drug treatments, cell viability was assessed using cell counting kit-8 (CCK8) assay (Dojindo Molecular Technologies, Tokyo, Japan) as described before 29. After incubation with CCK8 for 2-4hr, the absorbance was measured at the wavelength of 450 nm. Each concentration was performed in triplicate and repeated at least in three independent experiments. The IC50 of BI 2536 was calculated by Graph Prism software (GraphPad-Prism Software Inc., San Diego, CA, USA).

### Clone formation assay

NB cells were trypsinized into single cell and plated in 6-well dishes at a density of 1000 cells per well. The following day, cells were treated with serial concentrations of BI 2536 for 48hr. Then the media were changed and cells were incubated for a period of 2 weeks. After incubation, cells were then fixed with methanol and stained with Crystal Violet stain solution (cat. C0121; Beyotime Biotechnology, Shanghai, China).

### Cell cycle analysis

NB cells were washed by PBS and then fixed with 70% ethanol overnight at 4°C. The fixed cells were permeabilized with 0.5% Triton X-100 for 10 min. Then, cells were washed, stained with propidium iodide (PI 1.5 µmol/L) (cat. P4170; Sigma-Aldrich, St. Louis, MO, USA) and 25 µg/ml RNase A at room temperature in the dark for 1 hr. Cell cycle phases were analyzed by flow cytometry on a Beckman Gallios™ Flow Cytometer (Beckman, Krefeld, Germany) and MultiCycle AV DNA analysis software (Verity Software House, Topsham, ME, USA).

### Caspase-3 activity assay

The Caspase-3 activity was determined according to the manufacturer's protocol of Caspase-3 Activity Assay Kit (cat. KGA203; KeyGEN BioTECH, Nanjing, China). Briefly, NB cells were lysed in cold lysis buffer (supplied with DTT) for 30min on ice. Then, the supernatant fraction was collected by centrifuging at 10000 rpm for 1 min. After that, 200μg cell lysate was incubated with Caspase-3 substrate in reaction buffer for 4 hr in the dark. Subsequently, the absorption values were measured at 405nm by a scanning multi-well spectrophotometer (Bio-Rad Model 550; Bio-Rad, Hercules, CA, USA). The level of Caspase-3 activity was calculated from optical density (OD) values compared with the control group (Reaction buffer as control).

### Apoptosis assay

Apoptosis assay was performed according to the manual of FITC-Annexin V apoptosis detection kit (cat. 556420; BD Biosciences, Franklin Lakes, NJ, USA) was performed as described before 30. Briefly, NB cells were treated for 24 hours with BI 2536 before harvesting. Then, cells were collected and washed twice with cold PBS and then resuspended in 1x Binding buffer. Subsequently, cells were stained for 15 min with Annexin V-FITC and PI at room temperature in the dark before flow cytometric analysis.

### Transmission electron microscopy

NB cells were treated with BI 2536 for indicated times. Then, samples were fixed in 2.5% glutaraldehyde. Then, sample preparation and images acquisition were consigned to Fucheng Biological technology Co., Ltd (Shanghai, China). Images were acquired using a JEM-1200 electron microscope (JEOL, Tokyo, Japan).

### Autophagy observation

SH-SY5Y cells were infected with lentiviral particles expressing a fusion protein mRFP-GFP-LC3 (cat. GM-1314L204H; Genomeditech, Shanghai, China). Three days post infection, positive cells were screened by incubating in growth medium with puromycin (1μg/mL) for 7 days. For autophagy observation, SH-SY5Y-LC3-II cells were grown on coverslips and treated with BI 2536 for indicated times. LC3 II puncta were examined by confocal microscopy (Olympus; Olympus Corporation, Tokyo, Japan).

### Real-time PCR array analysis

Samples for RNA extraction were submerged in 1 mL Trizol ((Invitrogen, Carlsbad, CA, USA). Total RNA was extracted according to the manufacturer's instructions. Reverse transcription was performed from 2 mg of total RNA as template, 500 ng of six random primers (Thermo Fisher Scientific, Inc, Rockford, IL, USA), 200U of M-MLV Reverse transcriptase (Thermo Fisher Scientific), and 20U of RNase inhibitor (Thermo Fisher Scientific) in a total volume of 25µL. A Light cycler 480 Real Time System (Roche, Penzberg, Germany) was applied for quantitative real-time polymerase chain reaction analyses. Real-time PCR array (SABioscience Human Apoptosis PCR Array PAHS-3012, Frederick, MD, USA) analysis was performed in a total volume of 20 μL including 2 μL of cDNA template, 0.25 μM of each primer, and 10 μL of 2×SYBR Green mix (Roche). Gene expression quantification was determined by 2^-ΔΔCt^ method. Statistical significance of gene expression was calculated with the *t*-test using SPSS 11.5 software (Chicago, IL, USA).

### Western blot analysis

Western blot was carried out as described previously^30^. Cells were lysed in lysis buffer containing a protease inhibitor and a phosphatase inhibitor cocktail (Roche) for 30min. The supernatant was collected as whole cell lysates and protein concentration was quantified using the BCA Kit (Thermo Fisher Scientific). 25 to 50 μg denatured protein was separated by SDS-polyacrylamide gel followed by transferring onto PVDF membranes (Millipore, Bedford, MA, USA). Membranes were blocked in 5% skim milk for 1 hr and then probed with primary antibodies at 4°C overnight. After washing three times by TBST, the blots were incubated with corresponding secondary antibody at 37 °C for 1 hr. Finally, the bands were visualized by ECL detection kit (Pierce, Rockford, IL, USA) using LAS 4010 (GE Healthcare Life Sciences, Little Chalfont, UK).

### Antibodies

The primary antibodies were purchased from Cell Signaling Technology (cleaved-Caspase 3 (Cat: 9664, 1:1000), PARP (Cat: 9542, 1:1000), AMPKα (Cat: 5831, 1:1000), Phospho-AMPKα (Thr172) (Cat: 4188, 1:1000)) and Abcam Trading (Shanghai) Company Ltd. (PLK1(Cat: ab17056, 1:1000), LC3Ⅱ (Cat: ab48394, 1:1000), P62 (Cat: ab56416, 1:1000)). The antibody against β-actin, which was used as the reference protein, was purchased from Sigma-Aldrich (Cat: A5441, 1:5000). The horseradish peroxidase- conjugated secondary antibodies Peroxidase AffiniPure Goat Anti-Mouse IgG (H+L) (Cat: 115-035-003) and Goat Anti-Rabbit IgG (H+L) (Cat: 111-035-003) were purchased from Jackson ImmunoResearch Laboratories, Inc.

### Statistical analysis

All experiments were independently performed in triplicates at least 3 times. Statistical analyses were performed using GraphPad Prism version 5.0 (GraphPad Software, Inc., La Jolla, CA, USA). P values less than 0.05 were regarded as statistically significant (*P<0.05, **P<0.01, ***P<0.001).

## Results

### PLK1 was highly expressed in neuroblastoma cells

**Figure 1:**
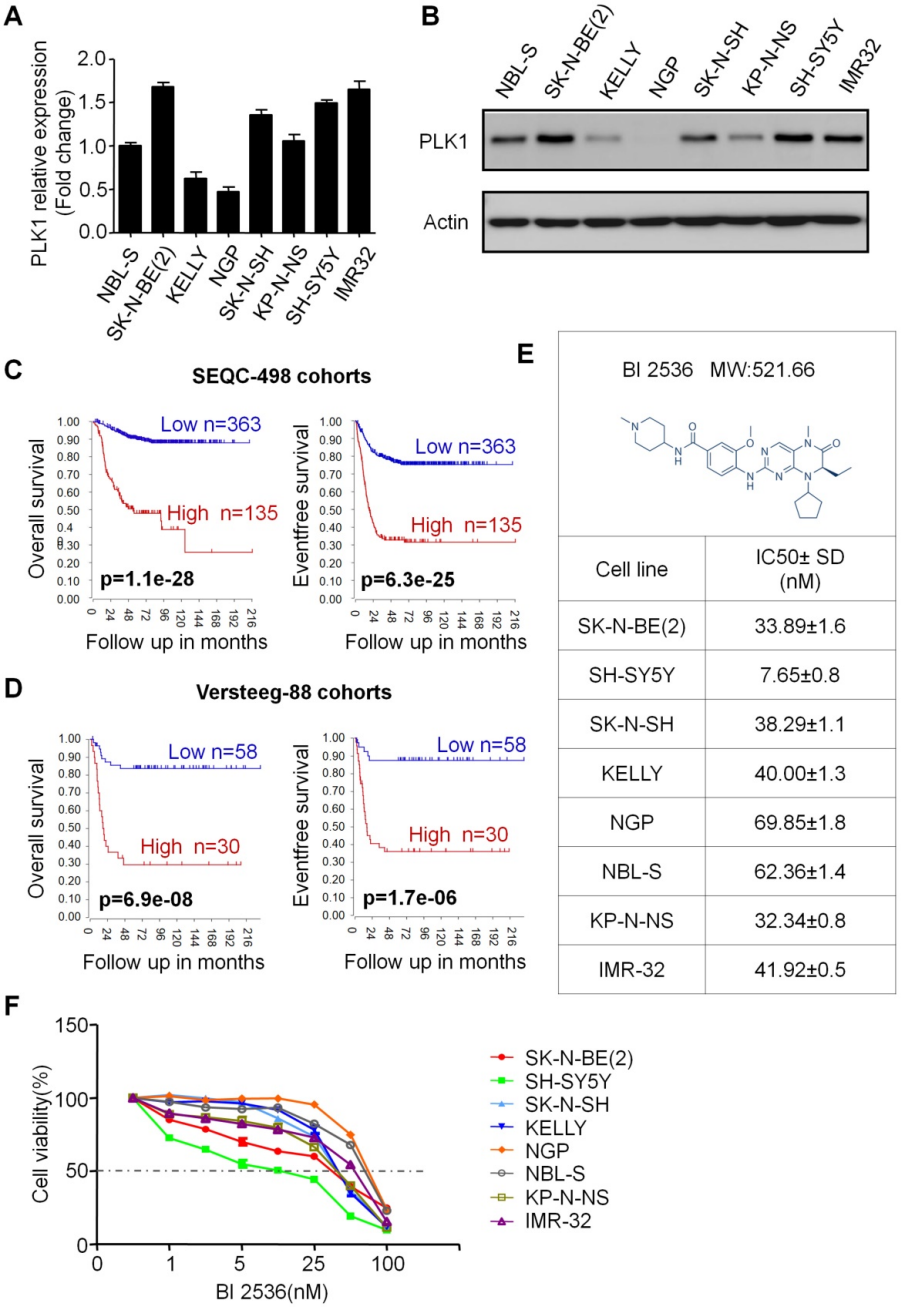
** PLK1 was over-expressed and inhibition of PLK1 by BI 2536 reduced viability in neuroblastoma cell lines. (A)** Quantification of PLK1 mRNA expression of neuroblastoma cell lines. **(B)** Western blot analysis of PLK1 expression in neuroblastoma cell lines. **(C)** Overall survival and event free survival plot generated from SEQC-498 cohorts in R2: Genomics Analysis and Visualization Platform (http://r2.amc.nl). **(D)** Overall survival and event free survival plot generated from Versteeg-88 cohorts in R2: Genomics Analysis and Visualization Platform (http://r2.amc.nl). **(E)** Molecular structure of BI 2536 and IC50 value of BI 2536 in neuroblastoma cell lines. The IC50 values were derived after plotting proliferation values on a logarithmic curve. Experiments were performed in quadruplicate and repeated twice. **(F)** Proliferation rate of neuroblastoma cell lines treated with BI 2536. NB cells (2 × 10^4^) were seeded in 96-well plates overnight and incubated with DMSO or increasing concentrations of BI 2536 (1, 2.5, 5, 10, 25, 50 or 100nM) for 24 h. Cell proliferation rate was calculated as a percentage of the DMSO treated control wells.

First, to assess the PLK1 expression in NB cells, we used MYCN amplified (NGP, KELLY, SK-N-BE(2), NGP and KP-N-NS) and non-amplified (SH-SY5Y, SK-N-SH and NBL-S) NB cell lines for real-time and Western blotting (Figure 1A&B). In accordance with Ackermann S *et al* reported before 28, our results showed that PLK1 is highly expressed in almost all of the 8 neuroblastoma cell lines except NGP cells. In addition, the status of MYCN amplified or not does not seem to affect PLK1 expression. Next, to evaluate whether PLK1 could be regarded as a potential therapeutic target in NB, we analyzed PLK1 mRNA transcripts in neuroblastoma tumor samples by using the R2: Genomics Analysis and Visualization Platform (http://r2.amc.nl). R2 is a web-based microarray and RNA-seq database which contains a large amount of data sets publicly available. In SEQC-498 cohorts containing 498 neuroblastoma patients' samples, high PLK1 expression (>median) was remarkable associated with both poor relapse free and overall survival of patients (Figure 1C). Similar results were found in Versteeg-88 dataset including 88 neuroblastoma samples (Figure 1D), demonstrating that PLK1 could be served as a potential predictor in NB patients' outcome.

### BI 2536 inhibits cell proliferation of neuroblastoma cells

**Figure 2:**
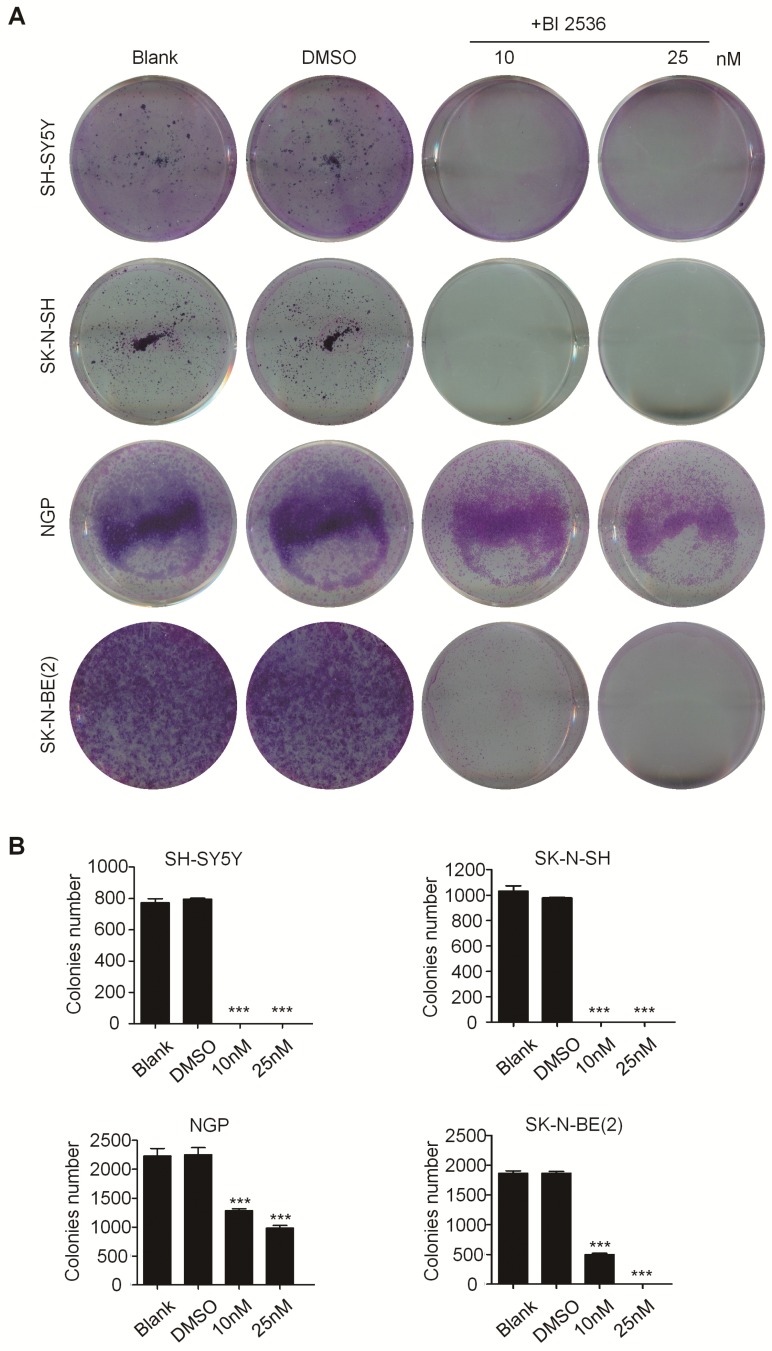
** BI 2536 inhibited clone formation ability in neuroblastoma cell lines. (A)** Clone formation assay of SH-SY5Y, SK-N-SH, NGP and SK-N-BE(2) cells incubated with DMSO or different concentrations of BI 2536(10 or 25 nM) for 2 weeks. **(B)** Clones number of SH-SY5Y, SK-N-SH, NGP and SK-N-BE(2) cells incubated with indicated concentration of BI 2536 or DMSO. *P<0.05, **P < 0.01 and ***P < 0.001. *P* values were determined by two-tailed t tests. All data are representative of three independent experiments with n = 3-6 per group and are means ± s.e.m.

In order to evaluate the effect of PLK1 inhibition, BI 2536, a specifically pharmacological inhibitor of PLK1, was applied (Figure 1E). We treated a panel of NB cell lines with BI 2536 and evaluated cellular viability by CCK8 assay. As shown in Figure 1F, BI 2536 significantly reduced cell viability with escalating doses of BI 2536 treatment in all NB cell lines tested, with the half-maximal inhibitory concentration (IC50) in the nanomolar range (Figure 1E). Furthermore, to observe the long-term effect of BI 2536 on cell proliferation, we chose two MYCN- amplied NB cell lines (SK-N-BE(2) and NGP cells) and two MYCN non-amplied NB cell lines (SH-SY5Y and SK-N-SH cells) for clone formation assay. The results showed that cell colonies decreased significantly after BI 2536 administration (Figure 2A & B). Taken together, these results demonstrate that BI 2536 potently inhibits proliferation and viability of neuroblastoma cells.

### BI 2536 disturbs cell cycle progress in neuroblastoma cells

**Figure 3:**
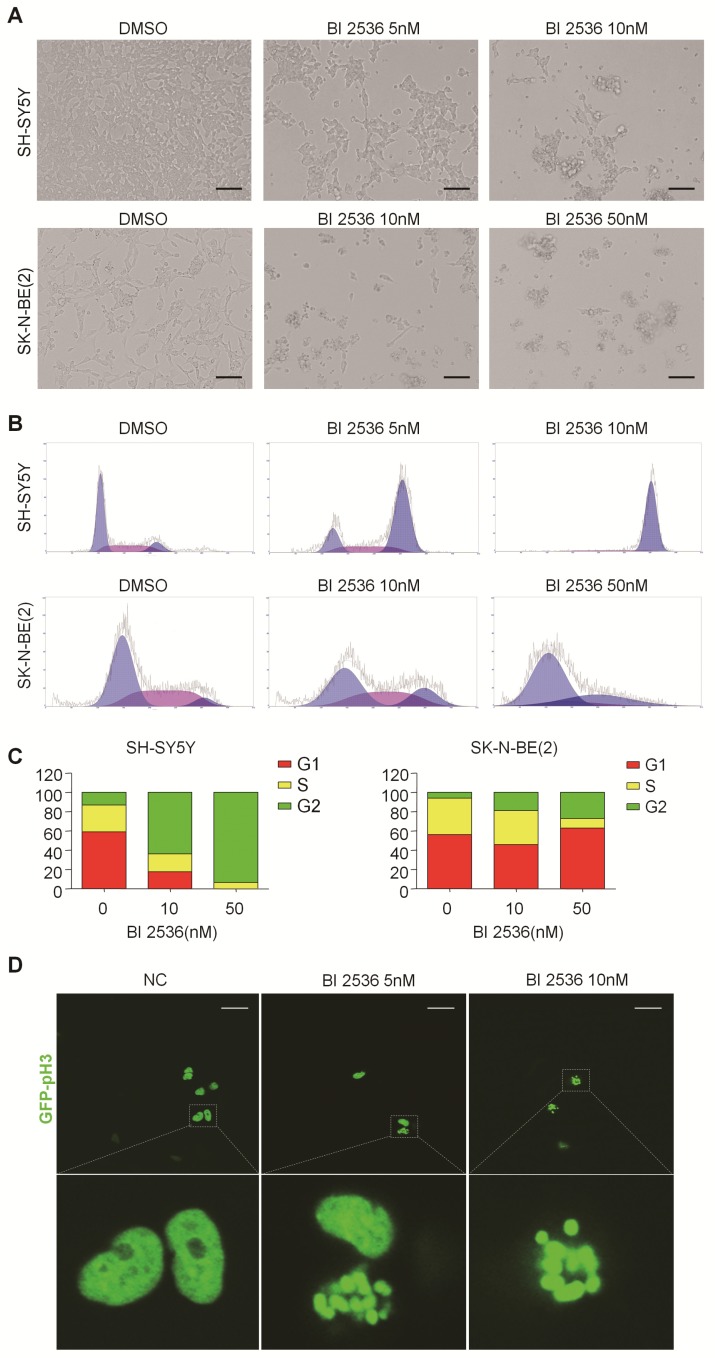
** BI 2536 induced cell cycle arrest in neuroblastoma cells. (A)** Photographs of SH-SY5Y and SK-N-BE(2) cells incubated with DMSO or BI 2536 for 24h. Scale bar represents 100 μm. **(B)** Cell cycle analysis showed SH-SY5Y and SK-N-BE(2) cells displayed cell cycle disorder after treatment with BI 2536. SH-SY5Y cells were harvested after 24h of treatment with 5 or 10nM BI 2536. SK-N-BE(2) cells are treated for 24 h with BI 2536 at 10 or 50nM compared with DMSO control mock treatment. **(C)** Proportion of the G2 phase cells increased significantly in both SH-SY5Y and SK-N-BE(2) cells after treated with indicated concentration of BI 2536. **(D)** Confocal analysis of GFP- Histone localization in SH-SY5Y cells. SH-SY5Y was infected by the GFP- Histone lentivirus at the multiplicity of infection (MOI) of 20 for 24h. After media replacement, cells were allowed to grow for another 48h. Then, BI 2536 was added into the cells for 24h before the confocal analysis. Scale bar represents 20 µm.

In particular, since BI 2536 showed the most pronounced anti-proliferation effects in SH-SY5Y and SK-N-BE(2) cells, we selected them for further studies. BI 2536 treatment resulted in significant cell morphology change, appearing as cell floating and shrinkage (Figure 3A). As PLK1 is part of the regulatory network controlling CDK1/cyclin B complex activity which controls entry into mitosis at the G_2_/M transition 31, we next examined the impact of BI 2536 treatment on cell cycle. Not surprisingly, cell cycle analysis displayed accumulation of cell populations in the G_2_ phase from 12.76±1.33% to 63.64±3.28% in SH-SY5Y cells in response to 5nM BI 2536 treatment for 24 hr. At the same time, a decrease in the population of G_1_ and S phase cells was observed. Higher concentration of BI 2536 administration induced more serious mitosis disorder. In similar, the G_2_ population was increased from 6.06±3.66% to 18.94±7.14%, with G_1_ fraction decreased from 56.30±4.63% to 46.01±4.54 % in SK-N-BE(2) cells exposed upon 10nM BI 2536 (Figure 3B & C). In addition, GFP- Histone was used to track the mitotic arrest. As shown in Figure (3D, in control group (SH-SY5Y cells treated with DMSO), GFP-Histone was dispersed in the nucleus, which means most cells are in interphase. However, after treated by 5nM BI 2536, some of the cells contained condensed GFP-Histone, indicating that these cells were arrested in pro-metaphase. More condensed GFP-Histone positive cells were present in the higher concentration group (10nM), suggesting BI 2536 can lead to pro-metaphase arrest in neuroblastoma cells. These data indicate that BI 2536 inhibits cell cycle progress by inducing G_2_/M phase arrest in neuroblastoma cells.

### BI 2536 induces apoptosis in neuroblastoma cells

**Figure 4:**
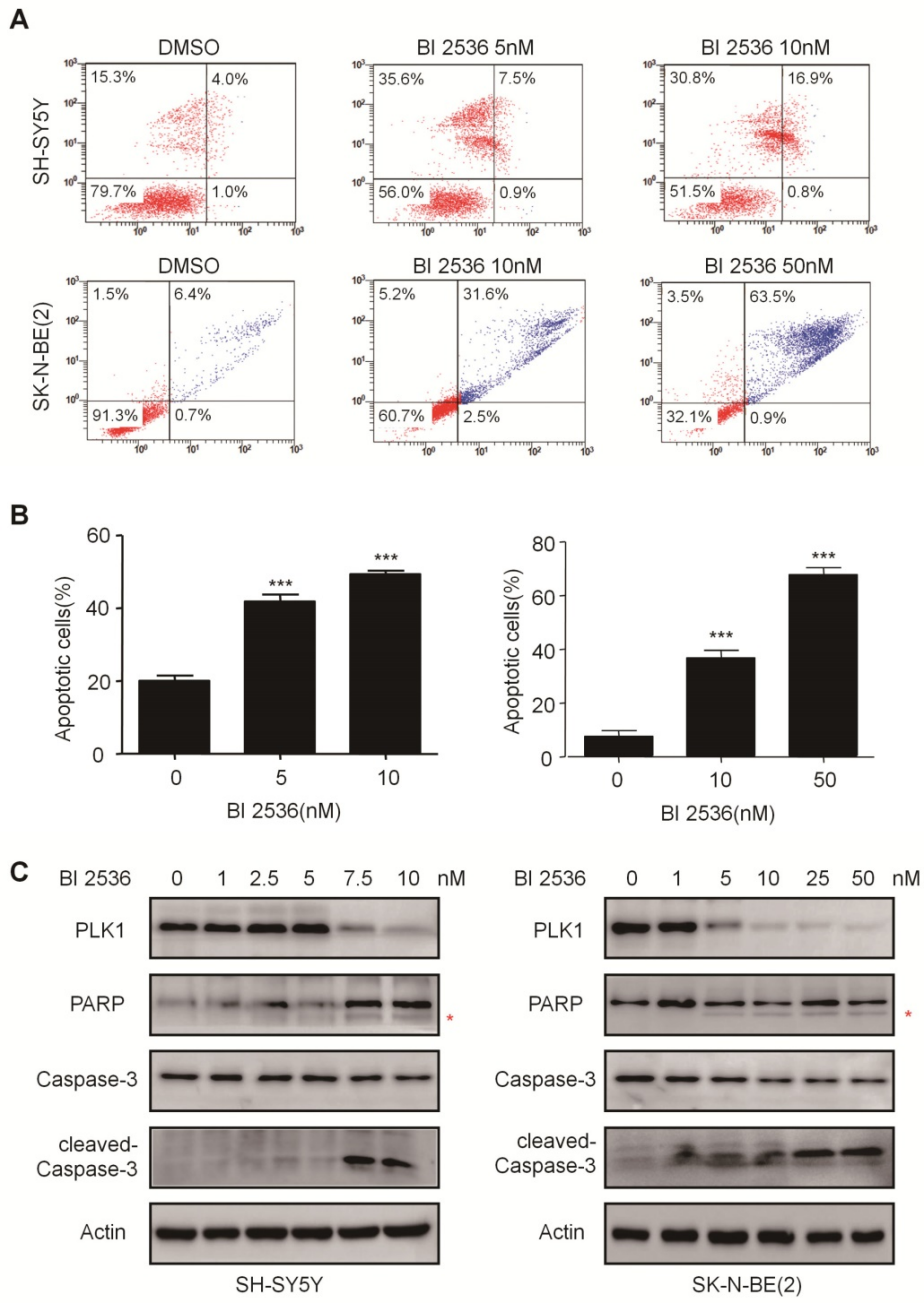
**BI 2536 induced cell apoptosis in neuroblastoma cells. (A)** Apoptosis analysis by Annexin V/PI staining in NB cells treated with BI 2536 for 24h. SH-SY5Y cells were harvested after 24h of incubation with 5 or 10nM BI 2536. SK-N-BE(2) cells were treated with BI 2536 at 10 or 50nM. **(B)** Proportion of apoptotic cells increased significantly in both SH-SY5Y and SK-N-BE(2) cells after treated with BI 2536 at indicated concentrations compared with DMSO control group. **(C)** Western blot analysis of PLK1, cleavage of PARP and Caspase-3 in lysates from SH-SY5Y and SK-N-BE(2) cells treated with serial concentrations of BI 2536 for 24h. * presents the cleaved band of PARP. *P<0.05, **P < 0.01 and ***P < 0.001. *P* values were determined by two-tailed t tests. All data are representative of three independent experiments with n = 3-6 per group and are means ± s.e.m.

To explore whether the anti-proliferation effect of BI 2536 is due to cell death, we investigated the induction of apoptosis using Annexin V staining in SH-SY5Y and SK-N-BE (2) cells treated with DMSO or BI 2536 for 24hr. As shown in Figure 4A & B, the proportion of apoptotic cell remarkably increased in the BI 2536 treated group (41.33±5.45 % in 5 nM treated group and 49.39±6.28 % in 10 nM treated group) compared with the control group (20.08±2.01%) in SH-SY5Y cells.

**Figure 5:**
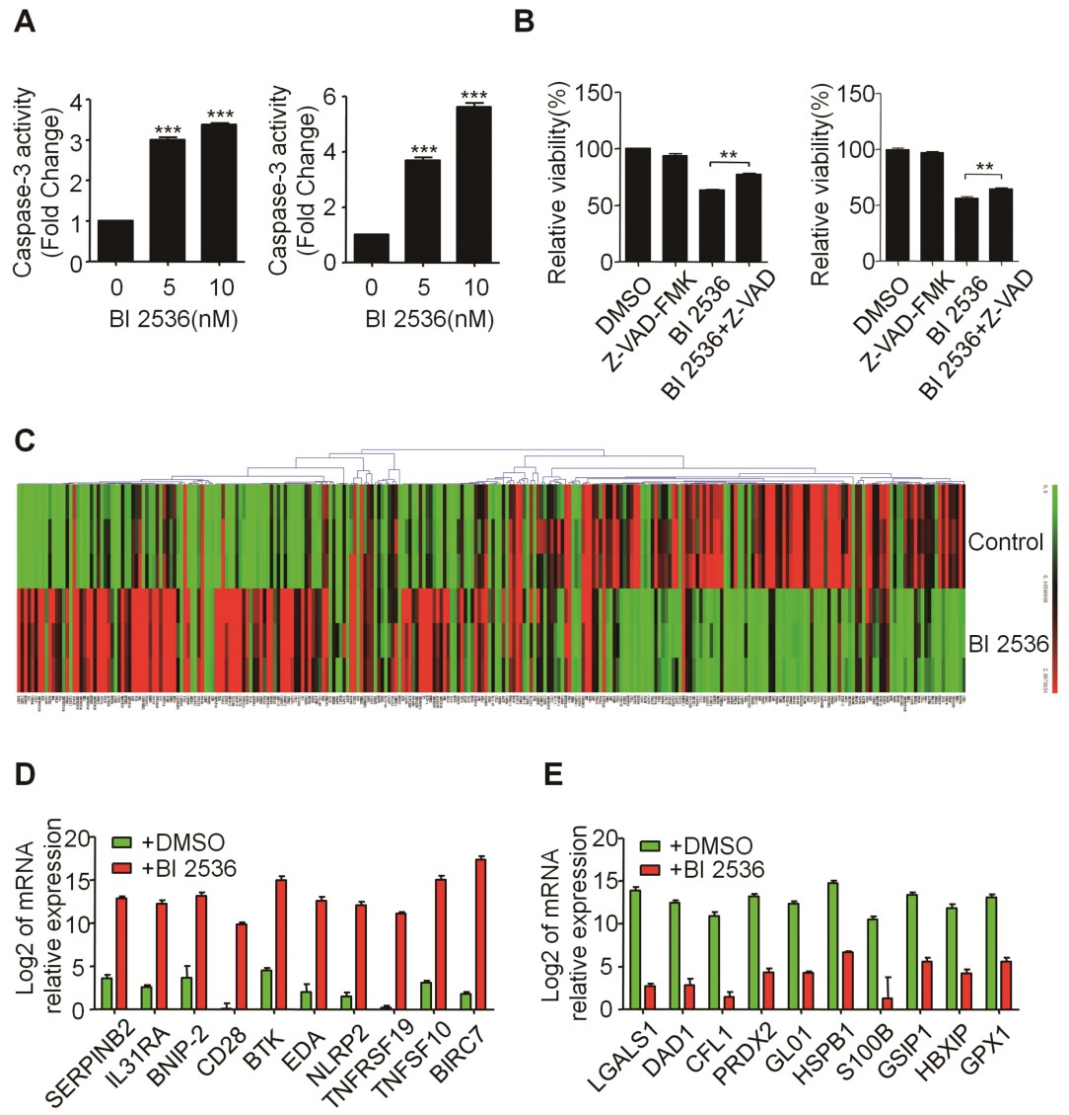
** BI 2536 affected the expression of apoptosis-related genes in neuroblastoma cells. (A)** Caspase-3 activity assay indicated BI 2536 treatment significantly increased Caspase-3 activity in both SH-SY5Y (left) and SK-N-BE(2) (right) cells. **(B)** CCK8 assay showed Z-VAD-FMK (25μM) partially reversed BI 2536-induced cell death in SH-SY5Y (left) and SK-N-BE(2) (right) cells. **(C)** Gene expression clustering of 370 key apoptosis-related genes in 10nM BI 2536-treated SH-SY5Y cells compared to DMSO-treated cells. **(D)** Transcript level of top 10 up regulated genes in SH-SY5Y cells treated with 10nM BI 2536. **(E)** Transcript level of top 10 down regulated genes in SH-SY5Y cells treated with 10nM BI 2536. *P<0.05 and ***P < 0.001. *P* values were determined by two-tailed t tests. All data are representative of three independent experiments with n = 3-6 per group and are means ± s.e.m.

The effect of BI 2536 on apoptosis induction was also observed in SK-N-BE (2) cells (Figure 4A & B). Furthermore, the classical apoptosis-associated markers---Caspase-3 and PARP were examined by western blot. An increased level of cleaved PARP and Caspase-3 was detected in BI 2536 treated cells. Furthermore, BI 2536 induced PARP and Caspase-3 cleavage in a dose dependent manner in both cell lines (Figure 4C). Also, to validate that the apoptosis induction effect of BI 2536 was caused by PLK1 inhibition, the expression of PLK1 was measured and the results showed that it gradually decreased on BI 2536 treatment (Figure 4C). We further evaluated the activity of Caspase-3 within cells. A dose-dependent increase in the Caspase-3 activity was observed in cells incubated with BI 2536 (Figure 5A). Administration of Z-VAD-FMK, a pan Caspase inhibitor, can reversed approximately 15 % of SH-SY5Y and 10 % SK-N-BE(2) cell viability, indicating that BI 2536-induced cell death was partially caspase-dependent (Figure 5B). These findings indicate that inhibition of PLK1 by BI 2536 effectively induces apoptosis in neuroblastoma cells.

### BI 2536 regulates apoptosis-related genes in neuroblastoma cells

To gain insight into the molecular mechanism underlying BI 2536-induced apoptosis, we applied SABioscience Human Apoptosis PCR Array PAHS- 3012Z to analyze which transcripts related to BI 2536 treatment. This real-time PCR array includes 370 key genes involved in apoptosis as we described before 29. We clustered the expression of 370 genes in DMSO or BI 2536 treated SH-SY5Y cells. The real time PCR array data showed that BI 2536 treatment significantly changed the gene profile by comparison with the DMSO group (Figure 5C). To be specific, we identified 99 genes upregulated (>5 fold) in BI 2536-treated group, such as BIRC7, TNFSF10, TNFRSF19 *et al* (Figure 5D). In addition, the expression of 79 genes were downregulated (>5 fold) after 24hr of BI 2536 treatment, such as LGALS1, DAD1, CFL1 et al (Figure 5E).

### BI 2536 attenuates autophagy in neuroblastoma cells

**Figure 6:**
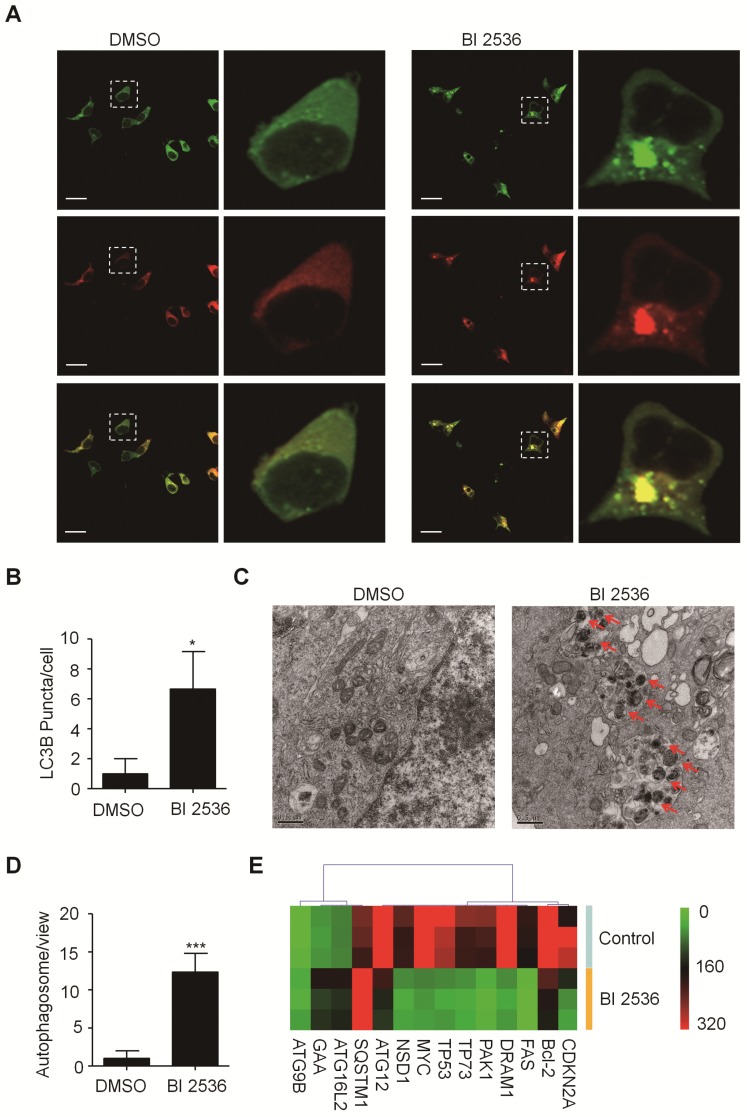
** BI 2536 attenuated autophagy in neuroblastoma cells. (A)** Immunofluorescence confocal images showing the increased formation of LC3-II puncta induced by BI 2536 treatment in SH-SY5Y cells. **(B)** Number of LC3-II puncta per cell in SH-SY5Y cells treated by DMSO or BI 2536. **(C)** Transmission electron microscopy images showing the increased formation of autophagosome induced by BI 2536 treatment in SH-SY5Y cells. The red arrows point out the autophagosome induced by BI 2536. Scale bar represents 0.5 μm. **(D)** Number of autophagosomes in every view in SH-SY5Y cells treated by DMSO or BI 2536. **(E)** Differentially Expressed autophagy-related genes in SH-SY5Y cells upon 10nM BI 2536 treatment. *P<0.05 and ***P < 0.001. *P* values were determined by two-tailed t tests. All data are representative of three independent experiments with n = 3-6 per group and are means ± s.e.m.

**Figure 7:**
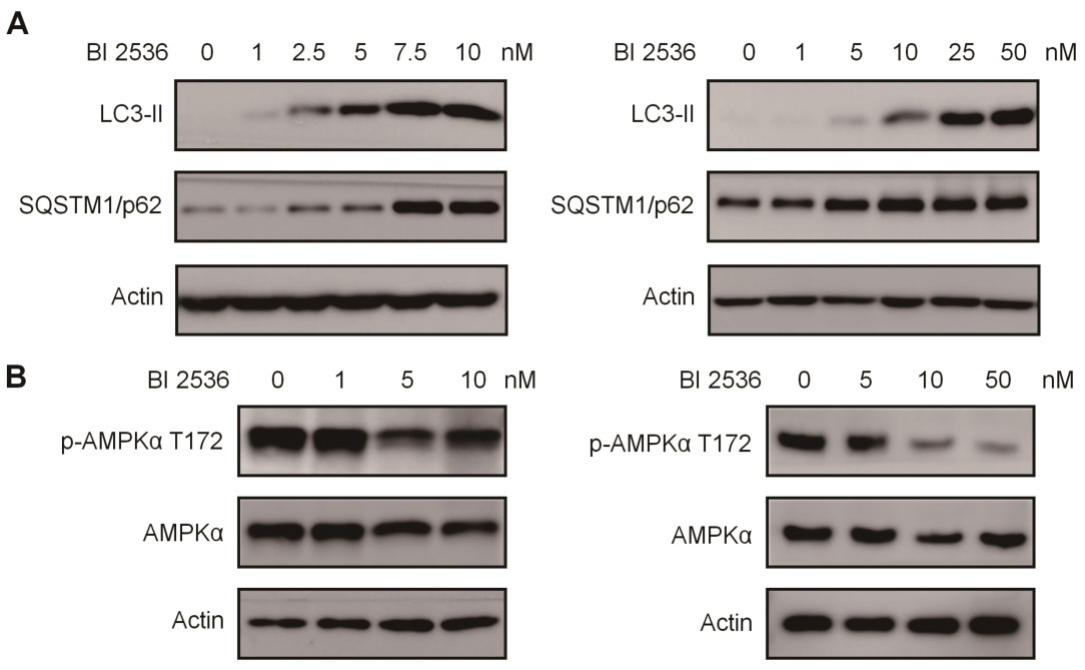
** BI 2536 mitigated autophagy by inactivating AMPK signaling. (A)** Western blot analysis of LC3-II, SQSTM1/p62 protein level in SH-SY5Y (left) and SK-N-BE(2) (right) cells after 24hr treatment with serial concentrations of BI 2536. **(B)** Western blot analysis of phosphorylation of Thr172 AMPKα in SH-SY5Y (left) and SK-N-BE(2) (right) cells incubated with BI 2536 for 24hr.

Given that PLK1 has been reported to promote autophagy by inhibiting the mammalian target of rapamycin complex1 (mTORC1) 32, we then sought to determine the effects of BI 2536 on autophagy. We established SH-SY5Y-LC3-II cells which stablely expressing a fusion protein mRFP-GFP-LC3-II. For autophagy observation, SH-SY5Y-LC3-II cells were treated with BI 2536 for 4 hr and the autophagy flux was observed by confocal microscopy. The results showed that BI 2536 significantly induced formation of cytoplasmic LC3-II puncta in SH-SY5Y- LC3-II cells (Figure 6A&B). Moreover, the transmission electron microscopy results revealed an increased number of autophagosomes in BI 2536-treated SH-SY5Y cells compared with control group (Figure 6C&D). Then, we detected several autophagy-related genes expression in SH-SY5Y treated by 10nM BI 2536 (Figure 6E). In addition, the Western blot analysis revealed that pharmacologic inhibition of PLK1 by BI 2536 treatment resulted in an increasing level of LC3-II in both cell lines. However, the expression of SQSTM1/p62, a marker of the maturation of the autolysosome, increased gradually as the concentration of BI 2536 increased in SH-SY5Y cells, suggesting that the process of lysosomal fusion was blocked by BI 2536 (Figure 7A). These results demonstrate that BI 2536 blocks the autophagic flux in neuroblastoma cells.

### BI 2536 regulates autophagic signaling in neuroblastoma cells

To further investigate the mechanism potentially involved in BI 2536-regulated autophagy, we utilized an autophagy real-time PCR array to identify genes regulated by BI 2536 treatment. This autophagy real-time PCR array contained 84 genes related to autophagy, which could be divided into two categories that are genes involved in autophagy machinery components and genes involved in regulation of autophagy. 4 up- and 10 downregulated genes induced by 4 hr of BI 2536 treatment were identified. The expression of ATG9B, GAA, ATG16L2 and SQSTM1 were up regulated in SH-SY5Y cells following incubation with BI 2536. Meanwhile, several PLK1 targeting genes, such as CDKN1A, Bcl-2 and MYC, were down regulated with PLK1 inhibition. Adenosine 5'-monophosphate (AMP)-activated protein kinase α (AMPK α) is a key regulator of autophagy process via suppression of mTORC1 or activation of ULK1 complex 33. Considering that phosphorylation of Thr172 of AMPKα is required for its activation, we next investigated the Thr172 phosphorylation status of AMPKα. As shown in Figure 7B, the phosphorylation of AMPKα Thr172 deduced in response to BI 2536 treatment in both SH-SY5Y and SK-N-BE(2) cells, suggesting the silence of AMPKα signaling. These data indicate that BI 2536 attenuates the autophagy process by inactivation the AMPKα signaling pathway.

## Discussion

There is now a growing body of evidence showing that PLK1 has attractive therapeutic potential in treatment of various types of cancers, including neuroblastoma. Our current study demonstrated that PLK1 was highly expressed in neuroblastoma cells, and its expression level was independent of the MYCN status. Furthermore, by virtue of public neuroblastoma datasets, we showed that high expression of PLK1 was closely related to the prognosis outcome of neuroblastoma patients. This observation is consistent with previous publications which reported the expression and prognosis value of PLK1 in NB patients 34. As PLK1 was reported over expressed especially in high risk NB, it represents a promising strategy against NB 28. Another group identified PLK1 as a critical signaling pathway required for NB tumor initiating cells' survival 35. All the above reports and our results indicate that PLK1 is a potential prognosis marker of neuroblastoma independent of MYCN copy number status.

With the development of PLK1 inhibitors, several PLK1 inhibitors displayed encouraging effect on diverse tumor types by suppressing tumor cell growth 36. BI 2536, which inhibits the enzymatic activity of PLK1 with an IC50 at nanomolar range, is a promising anti-tumor agent currently under clinical trial 37. In the present study, we evaluated the effect of BI 2536 on neuroblastoma cells. Inhibition of PLK1 by BI 2536 effectively exerted anti-proliferation effect and triggered apoptosis in a panel of neuroblastoma cell lines. Mitotic disorder occurred after BI 2536 administration in SH-SY5Y and SK-N-BE(2) cells, featured in G_2_/M phase arrest, which confirms the widely recognized crucial role of PLK1 in the precise regulation of cell division. These results were in line with a recent publication which reported that GSK461364, another PLK1 inhibitor, reduced cell viability, caused cell cycle arrest and induced apoptosis in preclinical *in vitro* and *in vivo* neuroblastoma model 38. The effect of BI 2536 on cell cycle disturbance has been also reported in different kinds of tumor cells, such as cervical adenocarcinoma 39, osteosarcoma 40 and glioblastoma 41. Our results provide further support to the *in vitro* and *in vivo* data, indicating that BI 2536 is a potent anti-tumor agent against neuroblastoma.

We explored the molecular mechanism of BI 2536-induced apoptosis by applying real-time PCR array and found BI 2536 altered large number of the expression of cell cycle and apoptosis-related genes, such as MYC, Bcl-2 and TNFSF10. The expression of SOCS2, which was found inhibited after PLK1 inhibition by RO3280 treatment in acute myeloid leukemia cells in our previous study 42, was also down regulated in neuroblastoma cells after PLK1 blockage, suggesting the involvement of SOCS2 in PLK1 pathway.

Importantly, we identified some novel genes which have not ever been reported to be regulated by PLK1. LGALS1 was the most significantly down regulated gene with PLK1 inhibition by BI 2536, suggesting it is possibly a downstream gene of PLK1. Galectins are a family of carbohydrate-binding proteins that participating in a variety of biological processes, such as cell adhesion, proliferation and apoptosis *et al* 43. The expression of LGALS1, also named galectin-1, was found to be associated with proliferation activity of human neuroblastoma 44, and high level of LGALS1 expression indicated poor prognosis of NB 45. Another recent study showed that knockdown of LGALS1 can suppress autophagy 46, as well as we observed in neuroblastoma cell by PLK1 inhibition. These evidences suggest that LGALS1 might serve downstream of PLK1 and contribute to the apoptosis induction and autophagy inhibition triggered by BI 2536 in neuroblastoma cells. DAD1, another downregulated gene under BI 2536 exposure, was once reported to play a role in cell survival under metabolism stress, in which the role of PLK1 was also reported 47. However, this study did not investigate whether there is a regulatory effect between DAD1 and PLK1. Mice deficient in DAD1 resulted in increased embryonic apoptosis 48. Additionally, we identified a lot of genes upregulated in response to BI 2536 treatment, in which BIRC7 was shown to be the most dramatically upregulated gene. BIRC7 is a member of the inhibitor-of-apoptosis protein (IAP) family of anti-apoptosis proteins. High level of BIRC7 expression was correlated with poor prognosis in neuroblastoma 49. In our study, increased BIRC7 expression might be a survival mechanism when tumor cells undergo the stimulation of pro-apoptosis agent. SERPINB2 is another upregulated gene in neuroblastoma cells following BI 2536 treatment. Increased SEPRINB2 expression is reported to be involved in nucleotide excision repair (NER) process 50. It is reasonable that BI 2536 treatment triggered the DNA damage repair process due to the apoptosis occurrence and SERPINB played a role in BI 2536-induced NER pathway. Here, we provide clues that the expression of the above genes might regulated by PLK1, and involved in BI 2536-induced apoptosis. Our results revealed the possible mechanisms involved in BI 2536-induced apoptosis in neuroblastoma cells.

Accumulating data suggest that PLK1 is involved in autophagy regulation. In this study, we demonstrated BI 2536 attenuated autophagy through blocking the autophagic flux. Although BI 2536 increased the LC3-II puncta formation, the protein level of SQSTM1/ P62 accumulated, indicating the process of autophagsome and lysosome fusion was blocked. These results were supported by a recent publication reported that PLK1 inhibited the activity of mTORC1 and promoted autophagy 32. Similar results were demonstrated by Valianou M *et al* that PLK1 inhibition promoted p62 protein accumulation 51. However, different from our observation, the LC3-Ⅰ expression decreased in their system. It probably due to the antibody we used was for LC3-II, but not LC3-I detection. Furthermore, we found that BI 2536 restrained autophagy through reduction of Thr172 phosphorylation of AMPKα, which is required for AMPKα activation. This finding can be explained by an early report that PLK1 and p-AMPKα^Thr172^ appeared to co-localized during mitosis, and PLK1 kinase activity is required for AMPKα activation at the mitotic apparatus 52. Whether PLK1 can directly phosphorylates AMPKα^Thr172^ still needs further study.

Meanwhile, we identified a cohort of autophagy- associated genes regulated by BI 2536. Autophagy is a highly conserved process that involving regulation of a variety of autophagy-associated proteins. DRAM1 (Damage-regulated autophagy modulator 1), is the key component that mediating the interaction between autophagosome and lysosome. In our study, BI 2536 treatment decreases the DRAM1 expression and attenuated the autophagy flux. The function of DRAM1 in autophagy flux was reported in a recent publication which showed DRAM1 plays important role in the interaction between autophagosome and lysosome. Overexpression of DRAM1 can restore the autophagy flux by inducing the conversion of autophagosome to autolysosome 53. Another autophagy related gene ATG12 is found down regulated after BI 2536 treatment. ATG12 is essential in early step autophagy. Cells lacking ATG12-ATG3 exhibit increased numbers of autophagosomes, indicating autophagy flux blockage 54. These disregulated genes might provide clues to explain the molecular mechanism of BI 2536-induced autophagy attenuation.

Taken together, the current study showed that PLK1 was over expressed in neuroblastoma cells. BI 2536, a specific PLK1 inhibitor, not only inhibited cell proliferation by disturbing cell cycle progress but also triggered cell apoptosis. Furthermore, BI 2536 blocked autophagic flux via suppression of AMPKα activation. This analysis provides evidence for the important role of PLK1 in neuroblastoma cell survival, and BI 2536 could be considered as a possible treatment strategy for neuroblastoma.
